# Enhanced lignin biodegradation by consortium of white rot fungi: microbial synergistic effects and product mapping

**DOI:** 10.1186/s13068-021-02011-y

**Published:** 2021-07-23

**Authors:** Tangwu Cui, Bo Yuan, Haiwei Guo, Hua Tian, Weimin Wang, Yingqun Ma, Changzhi Li, Qiang Fei

**Affiliations:** 1 School of Chemical Engineering and Technology, Xi’an Jiaotong University, Xi’an, 710049 China; 2 CAS Key Laboratory of Science and Technology On Applied Catalysis, Dalian Institute of Chemical Physics, Chinese Academy of Sciences, 457 Zhongshan Road, Dalian, 116023 People’s Republic of China; 3 Department of Chemistry, Pennsylvania State University, 215 Chemistry Bldg., University Park, PA 16802 USA; 4 Shaanxi Key Laboratory of Energy Chemical Process Intensification, Xi’an Jiaotong University, Xi’an, 710049 People’s Republic of China

**Keywords:** White rot fungi (WRF), Lignin biodegradation, Laccase, Manganese peroxidase, Synergistic effect, Product mapping

## Abstract

**Background:**

As one of the major components of lignocellulosic biomass, lignin has been considered as the most abundant renewable aromatic feedstock in the world. Comparing with thermal or catalytic strategies for lignin degradation, biological conversion is a promising approach featuring with mild conditions and diversity, and has received great attention nowadays.

**Results:**

In this study, a consortium of white rot fungi composed of *Lenzites betulina* and *Trametes versicolor* was employed to enhance the ligninolytic enzyme activity of laccase (Lac) and manganese peroxidase (MnP) under microbial synergism. The maximum enzymatic activity of Lac and MnP was individually 18.06 U mL^−1^ and 13.58 U mL^−1^ along with a lignin degradation rate of 50% (wt/wt), which were achieved from batch cultivation of the consortium. The activities of Lac and MnP obtained from the consortium were both improved more than 40%, as compared with monocultures of *L. betulina* or *T. versicolor* under the same culture condition. The enhanced biodegradation performance was in accordance with the results observed from scanning electron microscope (SEM) of lignin samples before and after biodegradation, and secondary-ion mass spectrometry (SIMS). Finally, the analysis of heteronuclear single quantum coherence (HSQC) NMR and gas chromatography–mass spectrometry (GC–MS) provided a comprehensive product mapping of the lignin biodegradation, suggesting that the lignin has undergone depolymerization of the macromolecules, side-chain cleavage, and aromatic ring-opening reactions.

**Conclusions:**

Our results revealed a considerable escalation on the enzymatic activity obtained in a short period from the cultivation of the *L. betulina* or *T. versicolor* due to the enhanced microbial synergistic effects, providing a potential bioconversion route for lignin utilization.

## Background

As the major component of lignocellulose, lignin has drawn great attention due to its aromatic structure characteristic that mainly composed of three phenylpropane units (i.e., sinapyl alcohol, coniferyl alcohol, and *p-*coumaryl alcohol). The utilization of lignin can be divided into the following two ways: direct application as soil amendments and pesticide sustained-release agents, or be used as a feedstock for the production of phenols and other high-value compounds [1]. The degradation of lignin into small molecules is still a challenge due to the complexity of linkages and recalcitrance by various bonds [2, 3]. Therefore, it is critical to explore efficient approaches to degrade lignin polymer via physical, chemical or biological routes [4]. As an example for funneling the mixture of monomers by chemo-catalytic methods to produce value-added products, in 2020, Liao et al. reported a biorefinery process that converts 78 wt% birch into xylochemicals, which represents one of the major breakthrough towards full utilization of lignin [5, 6]. In recent years, great efforts have been made on lignin biodegradation due to its mild condition, diversified choices of microorganisms, and high oxidative potentials. Mechanisms of the lignin degradation by major ligninolytic enzymes including laccase (Lac), manganese peroxidase (MnP), and lignin peroxidase (LiP) have been extensively studied. Among them, Lac is a polyphenol oxidase that is restricted to some lignin phenolic substrates due to its relatively low redox potential. The glycosylated heme protein MnP secreted by white-rot fungi was one of the most effective lignin degradation peroxidase [7]. The most common bonds in lignin including β-*O*-4, β–β, 4-*O*-5, and 5–5ʹ have all been found to be cleaved by biodegradation methods [8–11].

White rot fungi (WRF) plays an important role in biodegradation of lignin [12] and the catabolism in WRF have been thoroughly investigated in previous studies [13–15], of which the β-ketoadipate (β-KA) pathway has been identified as one of the most common pathways for biodegradation of lignin [16]. The products frequently derived from the process include monophenols, benzenediol derivatives, aromatic hydrocarbons, short-chain acids, etc. [17, 18]. *Lenzites betulina* and *Trametes versicolor* are two common WRF that can effectively secrete Lac and/or MnP to depolymerize lignin into small molecules [14, 19–23]. Although extensive studies of these two WRF have been carried out to investigate enzymes, mechanisms, and pathways of lignin biodegradation, to the best of our knowledgement, most of the reports only focus on monoculture of these fungus, which greatly limits the degradation rates of the bioconversion of lignin. On the other hand, lignin degradation by a consortium of fungi or bacteria has shown certain advantages in terms of improved enzymatic activities, novel secondary metabolites, better substrate utilization, higher enzyme diversity, etc. [24–26].

On the purpose of enhancing lignin biodegradation efficiency, studying the synergistic effects of fungi, and mapping the products from the degradation process [27, 28], herein a consortium of WRF composed of *L. betulina* and *T. versicolor* was investigated in batch cultures with lab-optimized medium. The enzymatic activities of two important ligninolytic enzymes (Lac and MnP) secreted by the WRF consortium were examined throughout the cultivation. The performance of lignin biodegradation was determined by calculating the degradation rates and running the scanning electron microscope (SEM) and secondary-ion mass spectrometry (SIMS). Furthermore, detailed product mapping was analyzed by employing heteronuclear single quantum coherence (HSQC) NMR and gas chromatography–mass spectrometry (GC–MS). Finally, a plausible degradation process via the catabolic pathways during the co-culture of *L. betulina* and *T. versicolor* was proposed based on experimental results.

## Results and discussion

### Synergistic effects of WRF consortium on ligninolytic enzyme activity and biodegradation

**Fig. 1:**
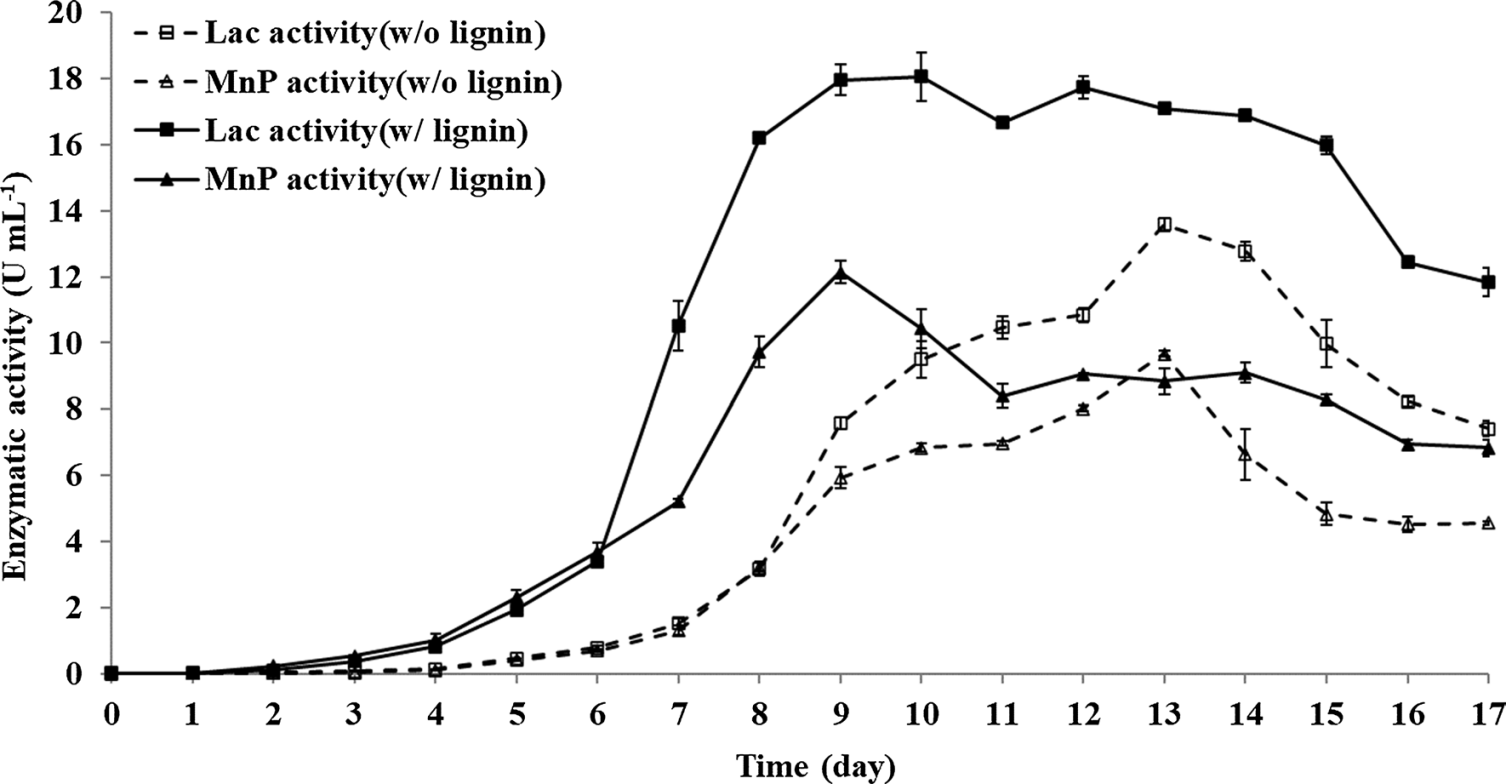
Time courses of Lac and MnP enzymatic activities in the culture of consortium. Dot lines present the cultures without adding lignin; solid lines present the cultures with adding lignin

The induction of ligninolytic enzymes was shown to be stimulated when culturing a consortium of WRF due to interspecific interactions [29–32]. Therefore, to investigate the synergistic influence of the WRF consortium on lignin biodegradation, enzymatic activities were analyzed daily during the cultivation with or without the supplement of lignin. As shown in Fig. 1, the highest enzymatic activities of Lac (18.06 U mL^−1^) and MnP (13.58 U mL^−1^) were achieved in the cultures in the presence of lignin, which reduced the produce time of ligninolytic enzymes over 30%. This finding may be explained that the interspecific interactions between WRFs accelerate a fungal metabolic switch of the formation from primary to secondary metabolites that stimulates the secretion of ligninolytic enzymes [33, 34]. It was believed that ligninolytic enzymes may be regulated differently at the interface of interspecific interactions in response to various stressful conditions. It has been reported that the competition of nutrients or the production of free radicals such as reactive oxidative species (ROS) derived from oxidative stress could cause stimulation to enzymatic inductions [35].

**Fig. 2:**
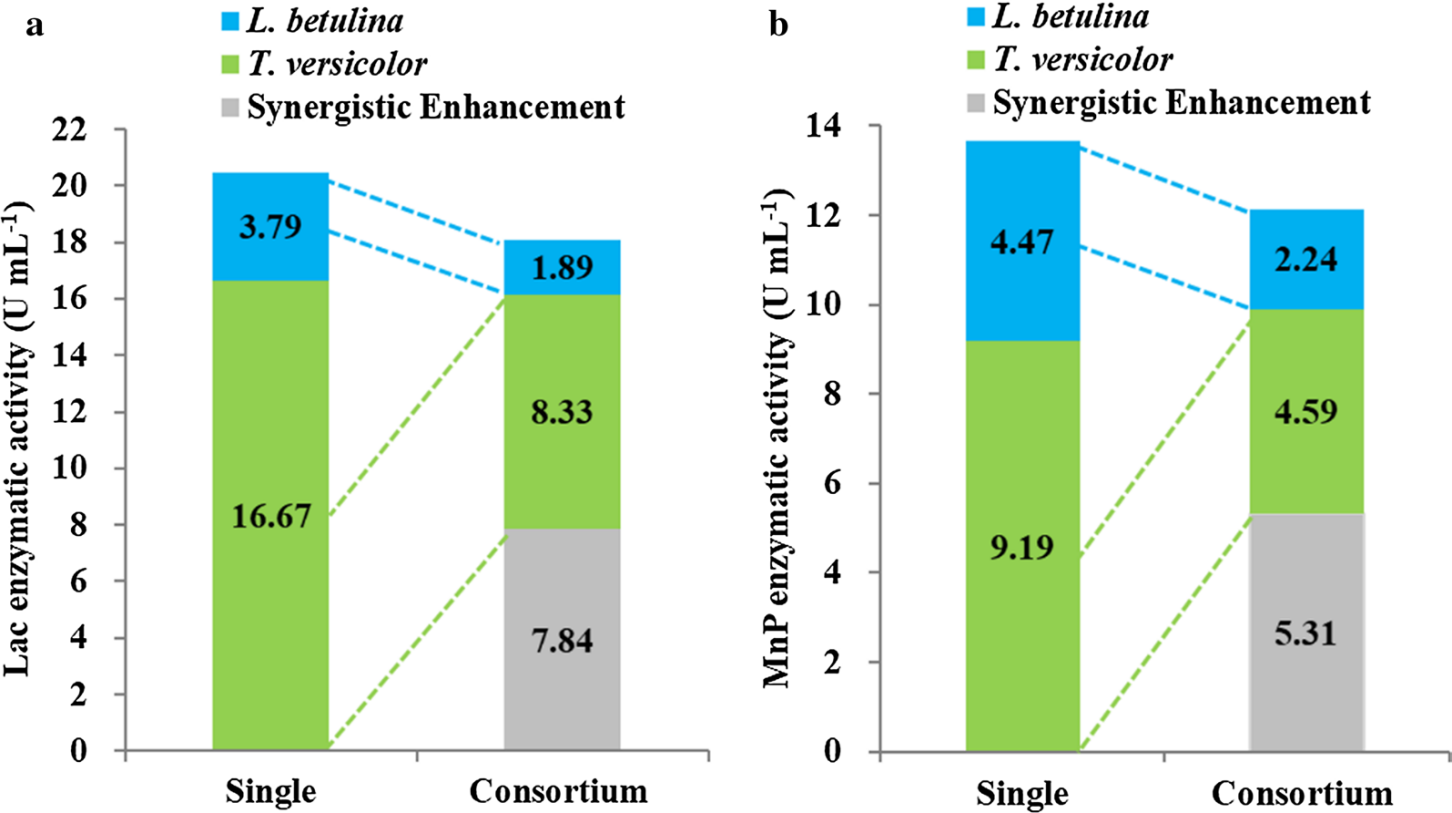
Enhanced synergistic effects on enzymatic activities of Lac (**a**) and MnP (**b**)

To have a comprehensive understanding on the advantages of consortium culture, the activities of Lac and MnP obtained in cocultures were compared with monocultures of *L. betulina* and *T. versicolor*. As shown in Fig. 2, the enzyme activities from cocultures were more than 40% higher than the sum of the values observed in mono-cultivation, mainly attributed to the fact that cocultivation of interacting fungi elevates expressions of Lac and MnP as well as the induction of novel isozymes. Ligninolytic enzymes such as Lac and LiP could be enhanced to mediate the stress and remove the ROS [32], which were approved by investigating the metabolites at the interaction zones, where novel metabolites were released in response to antagonistic interactions. It was obvious that the enzyme activities for both Lac and MnP obtained in the consortium cultures were higher than most of the previous reports with either monocultures or cocultures (Table 1), indicating an enhanced synergistic effect of the combination of *L. betulina* or *T. versicolor*, which promoted the inductions of ligninolytic enzymes. Nevertheless, the exact synergistic mechanisms of the paired WRFs need to be elucidated with more research data.

**Table 1:** Maximum enzymatic activities for Lac and MnP

Microorganism	Lac, U mL^−1^	MnP, U mL^−1^	References
*L. betulina*	0.026	0.03	[19]
*L. betulina*	0.127	0.039	[20]
*T. versicolor*	0.525	0.107	[20]
*T. versicolor*	0.37	0.0039	[21]
*T. versicolor*	0.159	0.109	[22]
*Inonotus obliquus*	–	159.0	[15]
Consortium of *Trametes* sp. AH28-2 and *Trichoderma* sp. ZH1	6.210	–	[32]
Consortium of *P. chrysosporium, T. versicolor, Aspergillus niger, Penicillium chrysogenum, Trichoderma harzianum,* and *P. citrinum*	–	66.70	[36]
Consortium of *Rhodotorula mucilaginosa* and *Pleurotus ferulae* JM301	10.58	–	[37]
Consortium of *Phanerochaete chrysosporium* Burdsall and *Trichoderma reesei* RUT-C30	< 0.6	2.39	[38]
Consortium of *L. betulina* and *T. versicolor*	18.06	13.58	This study

**Fig. 3:**
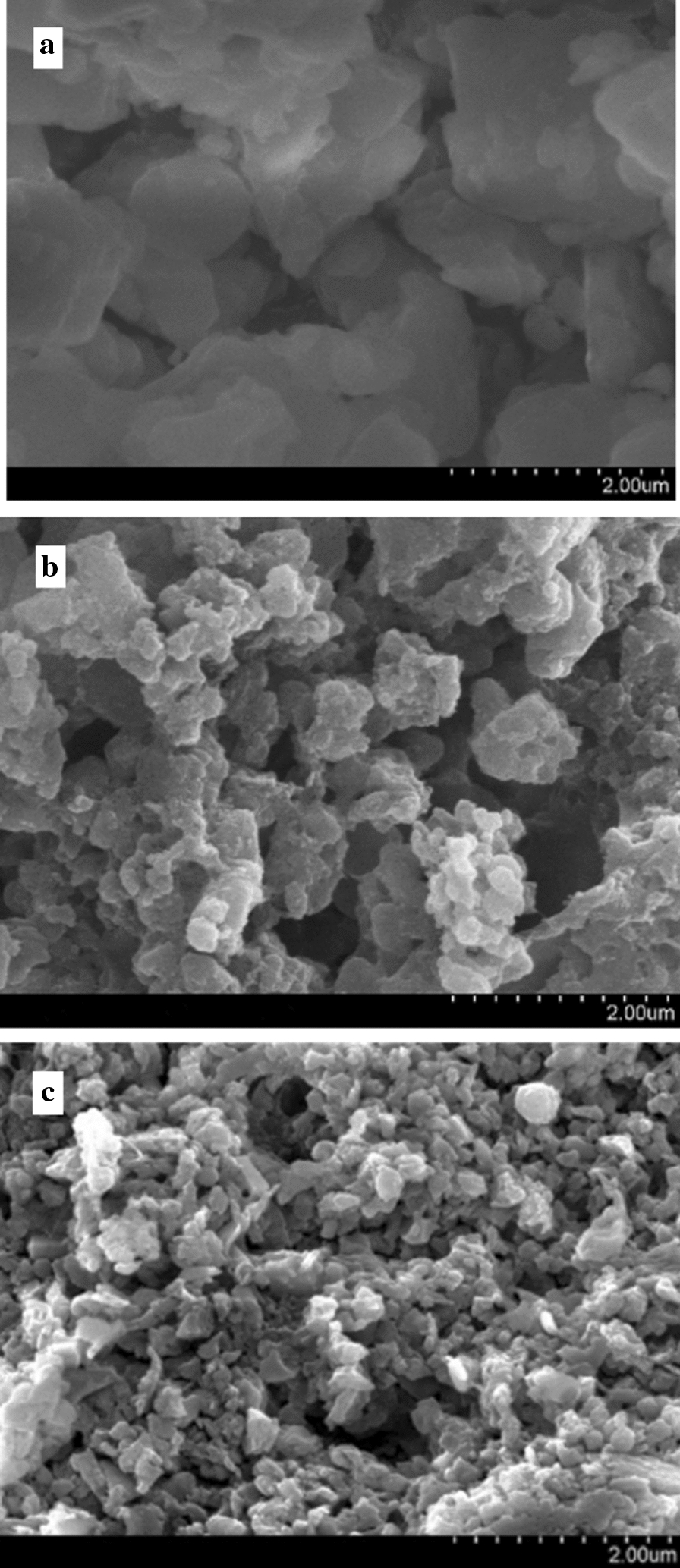
SEM images for comparisons of lignin samples before and after biodegradation. **a** Control sample; **b** lignin sample (with no microorganisms); **c** lignin sample from the consortium

Based on the enzymatic activities data under interspecific interactions, the influence of improved enzymatic activities on degradation products was studied. The results of degradation rates, in combination with SEM and SIMS results provided more insights into the biodegradation process. The lignin degradation rate of the consortium was determined to be 50% (wt/wt), which was much higher not only than that from the cultures of *L. betulina* (26.6%) and *T. versicolor* (37.2%), but also than previous reports with consortium of 34.1% (wt/wt) by *Dichomitus squalens* [39] or 28.37% (wt/wt) by microbial consortia [40]. Subsequently, SEM was performed on the purpose of identifying correlation of surface morphology with biodegradation (Fig. 3). The lignin after treatment by the consortium (Fig. 3c) showed that the particle size of lignin was reduced significantly to below 4 μm, which was smaller than the control sample (Fig. 3a) and the one incubated without microorganisms (Fig. 3b). In addition, more irregular fragments are observed in Fig. 3c, and the particles were more densely packed. This result was consistent with the report by Zhu et al. [41], in which a decrease in particle size was observed on lignin sample treated by *Bacillus ligniniphilus.*

### Product mapping of the lignin biodegradation by WRF consortium

**Fig. 4:**
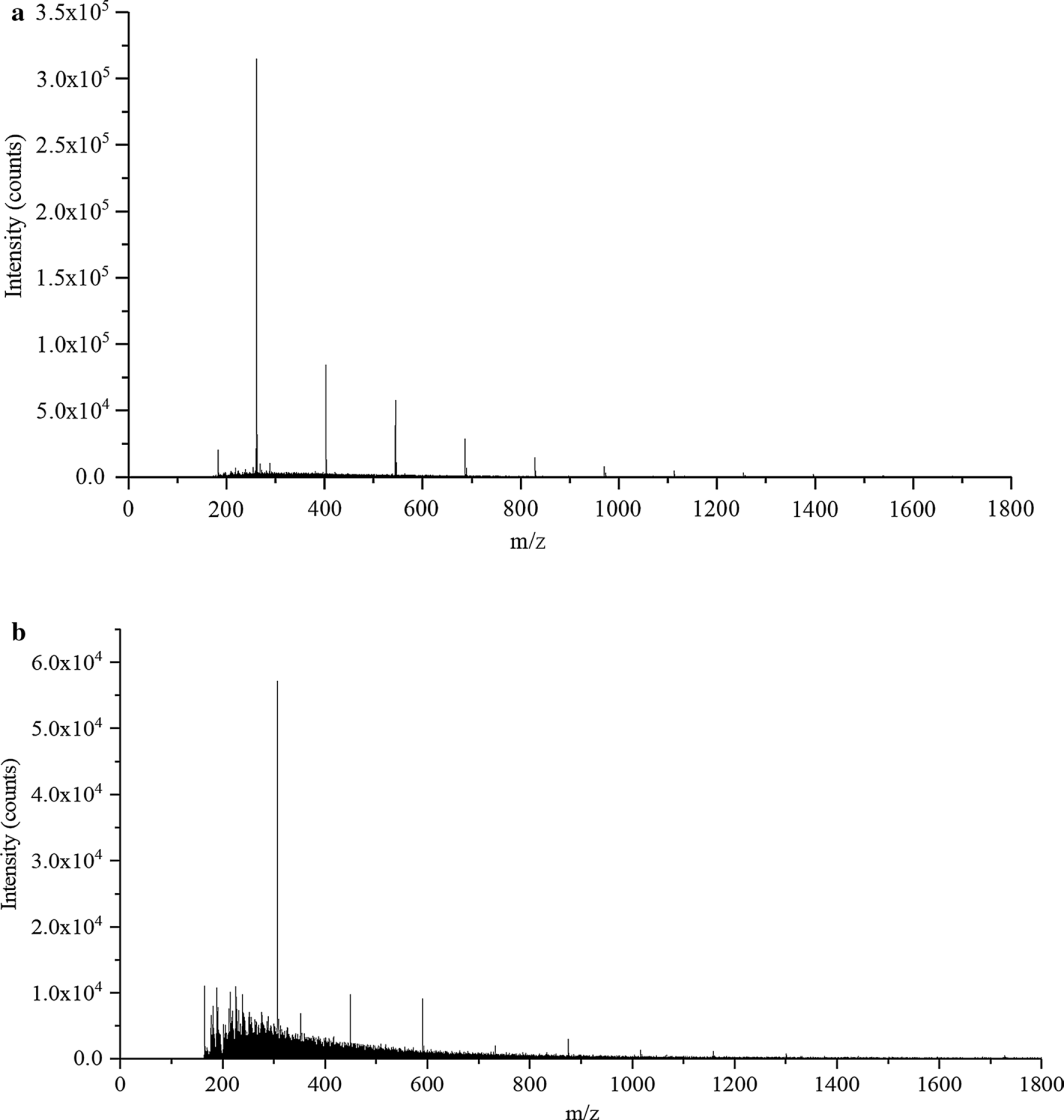
SIMS analysis of lignin for molecular weights comparisons. **a** Control sample; **b** lignin sample from the consortium

The degradation samples were analyzed with the aim to reveal the change of molecular weights of the products by SIMS, a state-of-art technique to analyze the topmost surface of the sample, which has shown immense potential for applications in various fields. The heterogeneous nature of lignin has evidently limited its solubility without pretreatment. However, pretreatment often leads to a certain degree of bond breakage in lignin sample, causing inaccuracies in the data to the original sample. SIMS resolved the above issues, since it was performed on solid samples, and gas cluster ion beam (GCIB) minimized the damage to lignin samples. In addition, the high energy GCIB employed offered chemical map of the sample surface at the spatial resolution of 1 micron. Figure 4 shows the SIMS spectra of the lignin sample before (control sample) and after the cultivation of the consortium with adding lignin. The signal intensities of the monomers at m/z 260 were approximately 5 times higher in the control sample than in the treated sample, indicating the cleavage of the linkages between the primary aromatic units after degradation. Many signals emerged for the sample after cultivation, ranging from approximately m/z 150 to 450, confirming that lignin macromolecules have been degraded into smaller fragments. From the above data, conclusions can be made that the consortium contributed to the improvement of the enzymatic activities and successful biodegradation based upon synergistic effects. Therefore, details of the products and possible pathways and mechanisms were explored by the following analyses.

**Fig. 5:**
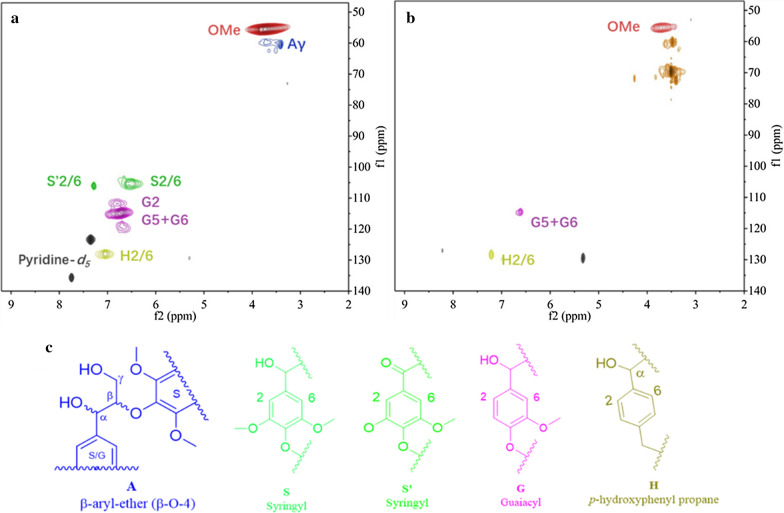
2D-HSQC NMR spectra of lignin. **a** Control sample; **b** lignin sample treated with the consortium; **c** corresponding lignin structures

The product mapping was conducted by HSQC and GC–MS, which provided a full picture of the products generated from the biodegradation of lignin and showed potential metabolic pathways and mechanisms. The structures of lignin analyzed by HSQC are shown in Fig. 5. The spectra of the control sample (Fig. 5a, δC/δH 50–140/2.0–9.0) could be divided into aromatic rings (δC/δH 90–140/6.0–9.0) and side chain regions (δC/δH 50–90/2.0–6.0). The region for aromatic rings showed multiple signals for Cα-oxidized syringyl (S'), *p-*hydroxyphenyl (H), guaiacyl (G), and syringyl (S) structures, which were the outcome of the natural route for lignin degradation cleaved by Lac and MnP [42]. The region for side chains showed methoxy (OMe) and β-O-4 structures (Aγ) and the solvent signal was assigned to Pyridine. Compared with the control sample, the signals for S and S' structures in the aromatic ring region and Aγ structures in the side chain region diminished (Fig. 5b). In addition, less signals for G and H moieties in the aromatic ring region and OMe moieties in the side chain regions were identified. Our findings are in a good agreement with previous literatures, where Lac was found to be responsible for the released aromatic subunits of lignin during biodegradation [42].

The signals at *ca.* (δC/δH 68–70/3.0–3.5) might be attributed to WRF consortium residue structure. The above results indicated that the fungi consortium preferentially removed the S moieties and underwent β-O-4 bonds breakage and cleavage of the aromatic rings, thereby depolymerizing lignin to produce small molecular products. The results for reduction of OMe groups and β-5 bonds breakage were in agreement with the report from Mao et al*.* [43]. In addition, Zhao et al*.* also reported an enzymatic and microbial synergistic degradation of lignin, and results also showed that β-O-4 subunits were preferentially removed [44]. These results suggested several possible mechanisms for lignin degradation, and will be further discussed with GC–MS results.

Products from lignin biodegradation were identified and characterized by GC–MS (Table 2). These products can be categorized into three groups including substituted aromatics, small molecule acids and aliphatic acids. First, substituted aromatics are side-chain cleavage products, which are also demonstrated by HSQC results. For example, 4-methylcinnamic acid (Table 2, Entry 21) came from degradation of coumaryl alcohol [45]. Second, *p-*hydroxybenzoic acid (Table 2, Entry 16) is a key intermediate of protocatechuic acid, both are typical intermediates in the protocatechuate branch pathway, and can be further metabolized via the β-KA pathway [45]. Similarly, benzoic acid (Table 2, Entry 4) came from degradation of cinnamic acid that leads to catechol, which is a key intermediate for the catechol branch of the pathway that goes into β-KA pathway [46]. Finally, oxalic acid, succinic acid, maleic acid, and other small acids were probably generated from ring opening via the *ortho* or *meta* oxidative ring fission [47, 48], which was in the agreement with the HSQC data, where the aromatic regions diminished after treatment. A final example is adipic acid (Table 2, Entry 13) [49] as a valuable dicarboxylic acid derived from muconic acid, which comes from the catechol *ortho* degradation pathway. In conclusion, the GC–MS data showed key intermediates for the side-chain fission and ring-opening, indicating that the β-KA pathway should be the main pathway for the lignin degradation. In addition, the upper funneling pathways including protocatechuate pathway and catechol pathway also possibly existed in the biodegradation system by the consortium.

**Table 2:**
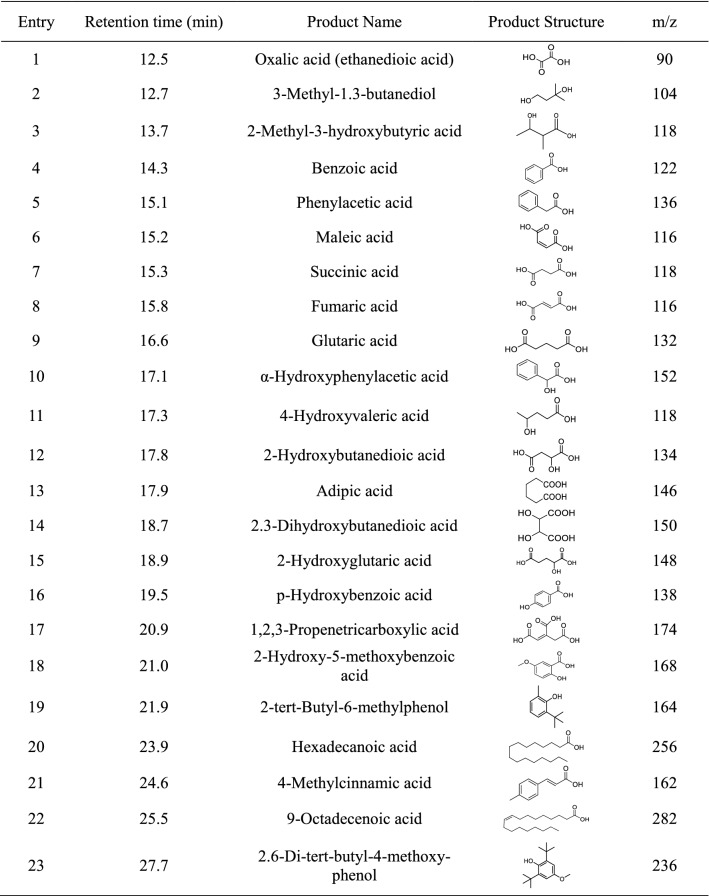
Lignin degradation products identified by GC–MS

## Conclusions

Lignin biodegradation by fungi consortiums shows great potential to overcome the bottlenecks of one single microorganism-catalyzed lignin degradation with regard to the low enzymatic activity, low enzyme diversity, and limited substrate scope. In this study, a considerable escalation on the ligninolytic enzymatic activities including Lac (18.06 U mL^−1^) and MnP (13.58 U mL^−1^) were obtained in a 30% shorten producing period from the cultivation of the WRF consortium relying on the microbial synergistic effects. These findings were confirmed by analyzing the surface morphology and molecular weights of the products by SEM and SIMS. Notably, the lignin degradation rate of the consortium was 40% higher than that from monocultures of *L. betulina* and *T. versicolor*. Moreover, 2D-HSQC NMR spectra exhibited the bond cleavage and diminish of the functional groups. Finally, the product mapping was identified by GC–MS and the possible pathway for lignin degradation was discussed. The present study provides an advantageous method for lignin biological treatment. Future research will focus on exploring the molecular mechanism of the synergistic effect of these fungi consortiums as well as converting lignin into value-added chemicals through the developed biological strategy.

## Materials and methods

### Microorganisms and culture conditions

The *L. betulina* and *T. versicolor* were purchased from China General Microbiological Culture Collection Center (CGMCC). These WRFs were maintained on potato dextrose agar (PDA) plates by a monthly subculture. The lab-modified culture medium (pH 5.4) used for all experiments consists of glucose (20 g L^−1^), peptone (5 g L^−1^), yeast extract powder (2 g L^−1^), KH_2_PO_4_ (2 g L^−1^), MgSO_4_ 7H_2_O (0.5 g L^−1^), MnSO_4_ (0.5 mM), CaCl_2_ 2H_2_O (0.1 g L^−1^), succinic acid (1.18 g L^−1^), ammonium tartrate (1.84 g L^−1^), thiamine (1 mg L^−1^), tween-80 (0.5 mL L^−1^), trace element (70 mL L^−1^) [50, 51]. The trace element components consist of MgSO_4_ 7H_2_O (3 g L^−1^), MnSO_4_ H_2_O (0.5 g L^−1^), NaCl (1.0 g L^−1^), FeSO_4_ 7H_2_O (0.1 g L^−1^), CoCl (0.1 g L^−1^), ZnSO_4_ 7H_2_O (0.1 g L^−1^), CuSO_4_ 5H_2_O (0.1 g L^−1^), KAl(SO_4_)_2_ 12H_2_O (0.01 g L^−1^), H_3_BO_3_ (0.01 g L^−1^), NaMo_4_ 2H_2_O (0.01 g L^−1^). The glass beans (id. 0.5 cm) were applied in all liquid cultures for WRF growth. The other chemicals were used without further purification unless otherwise stated. Lignin used in this study was pure sample without containing any cellulosic and hemicellulosic components, which was provided by Shandong Longlive Bio-Technology Co., Ltd. It belongs to industry alkaline lignin that was extracted from corn stalk. In detail, the raw corn stalk material (70 g) was first treated with 1 M NaOH at 50 °C for 3 h with the solid/liquid ratio of 1:20 (g/ml). After reaction, the filtrated liquid fraction was adjusted to pH 5–6 with 6 M HCl, and were then concentrated and precipitated in ethanol to remove hemicellulosic component. The aqueous ethanol filtrate containing lignin was concentrated under vacuum and reprecipitated by acidification with 6 M HCl to pH 2.0. Finally, the purified lignin was collected in 3.2 g after being dried at 50 °C in vacuum oven.

A loop of colonies of both strains from PDA plates was transferred in 250 mL flasks for seed cultures for 168-192hs. A 10% (vol/vol) inoculum was used for all experiments using 1000 mL flasks containing 600 mL medium. The seed solutions of *L. betulina* and *T. versicolor* of 6 mL were inoculated into the culture media. The inoculation solution of the consortium was made up of 3 mL of *L. betulina* and 3 mL of *T. versicolor*. Lignin of 1% (wt/vol) was added into the culture media to study the effect of lignin supplement on WRF growth. All the cultures were performed at 30 °C and 200 rpm in a rotary shaker (ZQZY-70CS, Zhichu, China) with triplicates.

### Enzymatic activity assays

Ligninolytic enzymatic activity assays were performed everyday using a UV spectrophotometer (Persee TU-1800, Beijing, China) in this study. The supernatant was collected from 1 mL culture solution after centrifugation at 8000 rpm for 10 min to remove the cells and residual lignin. Lac activity was determined by monitoring the reaction with ABTS as a substrate at 420 nm with reported procedures [52]. Lac activity was calculated employing Lambert–Beer law, the extinction coefficient (ε) is 36000 M^−1^ cm^−1^. The manganese peroxidase (MnP) activity was measured by monitoring the oxidation of 2,6-dimethoxyphenol (2,6-DMP) as the substrate at 470 nm (ε_470_ = 49,600 M^−1^ cm^−1^) [53]. The enzymatic activity was calculated with the UV absorption data according to reported methods [54].

### Determination of degradation rates

Degradation rates of lignin by employing a single and a mixture of fungi were determined by the Laboratory Analysis Protocol (LAP) from the National Renewable Energy Laboratory (NREL) [55]. In this method, the contents of acid-soluble and acid insoluble lignin need to be determined separately [56]. Before determining the lignin content, the lignin in the biomass needs to be dissolved under alkaline conditions and precipitated under acidic conditions [57]. The lignin degradation rates were determined combing the acid insoluble and soluble portions of the sample according to the NREL protocol.

### Scanning electron microscope (SEM)

The changes of morphology and structures were observed by SEM. The samples were prepared by centrifuging the culture solution at the end of cultivation at 8000 rpm for 10 min and the precipitate was collected and lyophilized for 48 h to achieve a constant weight. Samples were coated with gold powder by a spray meter beforehand [41]. The samples were subjected to analysis by a TESCAN MALA3 LMH, Czech Republic.

### Secondary-ion mass spectrometry (SIMS)

Lignin samples after cultivation were centrifuged for 10 min at 8000 rpm. The precipitate was placed at –80 °C for 24 h and then freeze dried under reduced pressure. The samples were then grinded into powders. The fungi cells were removed by adjusting the pH to 12 by addition of NaOH (2 mol L^−1^) and placed at 30 °C water bath for 24 h, then centrifuged at 5000 rpm, room temperature for 5 min. The supernatant was transferred and pH was adjusted to 3 by addition of H_2_SO_4_ (2 mol L^−1^), then placed in 30 °C water bath for 24 h. The final sample was obtained by centrifuging at 5000 rpm, room temperature and freeze dried again under reduced pressure for 24 h. The lignin sample without fungi was pressurized into a thin layer with a thickness of approximated 5 mm. The top surface ~ 100 nm of the sample was subjected to SIMS measurement with a novel gas cluster ion beam (GCIB) and J105 3D Chemical Imager (Ionoptika, UK) with published procedures [58].

### Heteronuclear single quantum coherence (2D-HSQC) NMR

Lignin cultivation samples were pretreated by centrifugation, lyophilization, alkaline dissolution, centrifugation, acid precipitation and re-lyophilization. Subsequently 50 mg sample was dissolved in 0.5 mL DMSO-D6 and the solvent peak was used as a reference. 2D-HSQC measurements were performed at 25 °C with a Bruker AVAVCE III HD 700 MHz spectrometer and the Bruker standard pulse program was used [59]. HSQC cross-signals were analyzed and assigned by comparison with published results.

### Gas chromatography–mass spectrometry (GC–MS)

Samples for GC–MS analysis were taken at 50 mL aliquots from the cultivation culture and centrifuged at 8,000 rpm for 10 min to remove the cells. The centrifugation condition was and the pH was adjusted to approximately 2 by addition of 6 mol L^−1^ HCl to the centrifuged supernatant. Subsequently, the mixture was derivatized according to reported procedures [60].The derivatized sample (1 μL) was injected into the GC–MS (Thermo Fisher Scientific Trace ISQ, America). The capillary column used was HP-5 with Helium as the carrier gas, and the solvent delay time was 180 s. The injection temperature, ion source temperature, and transfer line temperature were 280, 250, and 200 °C, respectively. The column temperature program was set as follows: first heated from room temperature to 50 °C for 5 min, then the temperature was raised at 10 °C min^−1^ acceleration rate to 300 °C, then maintained for 5 min. The ionization mass spectrum in the range of 30–550 m/z was recorded in Full Scan mode and 70 eV electron energy, and the results were compared with standard mass spectrometry databases to determine the products after lignin degradation [61].

## Data Availability

Not applicable.

## Abbreviations

Lac: Laccase
MnP: Manganese peroxidase
SEM: Scanning electron microscope
SIMS: Secondary-ion mass spectrometry
HSQC: Heteronuclear single quantum coherence
GC–MS: Gas chromatography–mass spectrometry
LiP: Lignin peroxidase
WRF: White rot fungi
β-KA: β-Ketoadipate
ROS: Reactive oxidative species
S': Cα-oxidized syringyl
H: *p-*Hydroxyphenyl
G: Guaiacyl
S: Syringyl
OMe: Methoxy
Aγ: β-*O*-4 Structures
PDA: Potato dextrose agar
2,6-DMP: 2,6-Dimethoxyphenol
GCIB: Gas cluster ion beam
